# Design strategies for VR science and education games from an embodied cognition perspective: a literature-based meta-analysis

**DOI:** 10.3389/fpsyg.2023.1292110

**Published:** 2024-01-08

**Authors:** Xiuyu Lin, Runbo Li, Zhirong Chen, Jiayi Xiong

**Affiliations:** School of Information Technology in Education, South China Normal University, Guangzhou, China

**Keywords:** embodied cognition, virtual reality, educational games, meta-analysis, strategy

## Abstract

**Introduction:**

Natural science education, as an important means to improve the scientific literacy of citizens, combines science education games with virtual reality (VR) technology and is a major developmental direction in the field of gamified learning.

**Methods:**

To investigate the impact of VR science education games on learning efficiency from the perspective of embodied cognition, this study uses the China National Knowledge Infrastructure (CNKI) and Web of Science (WOS) databases as the main source of samples. A meta-analysis of 40 studies was conducted to examine teaching content, game interaction, and immersion mode.

**Results:**

The study found that (1) VR science and education games have a moderately positive impact on the overall learning effect; (2) regarding teaching content, the learning effect of skill training via VR science and education games is significant; (3) regarding interaction form, the learning effect on active interaction is significantly better than that of passive interaction; (4) regarding immersion mode, somatosensory VR games have a significant impact on the enhancement of students’ learning; (5) regarding application disciplines, VR science education games have a greater impact on science, engineering, language and other disciplines; (6) regarding academic segments, the learning effect on college students is most significant; and (7) regarding experimental intervention time, short-term intervention is most effective.

**Discussion:**

Accordingly, this article proposes strategies for VR science game design from the perspective of embodied cognition: a five-phase strategy including skill training, human-computer interaction, and environmental immersion, aiming to improve the learning effect and experience of users.

## 1 Introduction

With the rapid development of digital technology, exploring ways to improve the quality of teaching by means of artificial intelligence (AI), virtual reality (VR) and other technologies has received focused attention. The K-12 Science Education Framework and the Next Generation Science Standards in the United States emphasize the importance of leveraging technology to enhance science education (National Research Council, 2011; National Research Council, 2013). These frameworks and standards aim to elevate students’ scientific literacy by cultivating innovation, critical thinking, and problem-solving skills through evidence-based inquiry processes. Notably, the Next Generation Science Education Standards explicitly introduce the concept of “scientific practices,” emphasizing that students learn science through practical experience and utilize technology for scientific exploration and data analysis. The General Office of the Ministry of Education of China (2023) issued a circular on the Deepening Action Program for the Reform of Basic Education Curriculum and Teaching, which emphasizes the enhancement of scientific literacy among students at the basic education stage, the strengthening of the teaching of science subjects and the sustained and in-depth development of popular science education. In the same year, the document “Opinions on Strengthening the Work of Science Education in Primary and Secondary Schools in the New Era” focused on improving the quality of science education, exploring the use of AI, VR and other technological means to improve and strengthen experimental teaching (Ministry of Education and Other Eighteen Departments, 2023). In addition, European education policies have also significantly propelled the advancement of science education. Taking the United Kingdom as an example, the Department for Education (2015) has underscored the importance of science education in its latest science curriculum standards. This emphasis aims to foster students’ interest in scientific inquiry, elevate their enthusiasm for learning science, and empower them to apply their acquired knowledge in their daily lives.

VR science education games based on the theory of embodied cognition provide theoretical guidance to promote the interaction between individuals and the virtual environment and realize the game teaching of sensory participation and physical and mental unity (Ma et al., 2014), providing theoretical support for VR educational games.

### 1.1 Embodied cognition

Influenced by Western “mind–body dualism,” traditional cognitive science understands human cognitive activity as an independent abstract symbolic arithmetic processing process of the human brain, and the body can only passively receive external information (Ye, 2010). In this regard, the French philosopher Merleau-Ponty first put forward the phenomenological theory of the body (perception), breaking the traditional dichotomy between mind and body.

First, the phenomenological theory of perception holds that the mind is embodied and that the body is not just a biological organ but an “active entity” (Fan and Zhao, 2020). Humans can interact with the objective world through both active and passive interactions and acquire new cognitions and skills in the process. In active interaction, the human being is the initiator of the interaction, interacting with the objective world through the active application of physical activities and sensory organs (seeing, hearing, touching, smelling). Passive interaction, on the other hand, is close to the human cognitive activity of traditional cognition, in which the human being mainly receives stimuli from the outside world and uses the human brain for information processing.

Second, the theory of embodied cognition emphasizes the “embodiedness” of cognition and considers the relationship between mind and body as the basic framework for understanding human existence and activity (Wang and Zheng, 2016) and that the production of new knowledge requires an effective interaction between the object of cognition, the perceiving subject and the environment (Zhang, 2016). Research has shown that embodied immersion has a positive effect on the cognitive performance of learners. Johnson-Glenberg (2018) used desktop immersion to teach physics concepts and found that students in the active group performed significantly better than those in the passive group. Junokas et al. (2018) used a gesture recognition system to design four different types of gestures to represent the four symbols of mathematical operations to facilitate personalized interfaces and multimodal learning scenarios.

Creating embodied learning environments based on modern technology is becoming an emerging field of research, and researchers are actively expanding the functions of human-computer interaction technology to build stable embodied learning environments based on human perceptual characteristics to make the learning process more in line with the cognitive law of physical and mental unity.

### 1.2 VR science and educational games

Serious games refer to games developed with the specific intent of providing players with an educational experience that is engaging and meaningful (Djaouti et al., 2011). Serious VR games can simulate real-life experiences that offer a high level of interactivity and realism (Alrehaili and Al Osman, 2022). VR educational games are a specialized form of virtual reality serious game, focusing on the educational domain, especially in science education.

With the development and maturation of VR games, there are currently many scholars in academia who are applying VR games to science-related disciplines and conducting empirical research. Shi et al. (2022) integrated knowledge of quadratic functions into the design of a game-based immersive VR learning environment. Following gameplay, students in the experimental group demonstrated a significant improvement in their math achievement and enhanced learning motivation, while the control group students did not exhibit any improvement in math achievement. Bouali et al. (2019) introduced a virtual reality (VR)-based learning game to support the teaching and learning of object-oriented programming (OOP) concepts in computational education. The game’s design allows players to visually grasp OOP concepts, including object creation, instantiation, and manipulation. Sung et al. (2018) found that in an elementary school geoscience course, students demonstrated better academic performance, problem-solving abilities, and deeper learning strategies and motivations when learning through 3D experiential games. Butt et al. (2018) used a game-based VR system designed to practice urinary catheterization. This system aimed to assist nursing students in acquiring and retaining fundamental skills essential for patient safety. The findings suggest that utilizing game-based VR for nursing skill practice may serve as an effective method for fostering mastery learning and long-term retention. Xiao (2023) applied virtual reality teaching resources to primary school science classroom teaching, and found that the average grades and pass rates of the experimental class were higher than those of the control class, and the learning interests and attitudes of the experimental class students were also improved.

China is facing a steady decline in enthusiasm for scientific enquiry among young people (Linder et al., 2007). Realistic VR game situations and various interactive means provide technical support to enhance the enthusiasm of young people for scientific exploration (Zhong and Chen, 2018). However, the application effect of VR games is still controversial, and some studies have pointed out that the effect of knowledge transfer in a virtual reality environment is not significantly different from that of traditional learning (Zhong and Chen, 2018).

On the one hand, the interactivity of existing VR games is insufficient. Some science educators tend to hold the view of “game instrumentalization,” copy the “teacher-center” mode of teaching, and overemphasize the richness of the playground in game design, neglecting the learning input and human-computer interaction process, which results in poor learning results (Macías-Díaz, 2008; Jiang et al., 2021). On the other hand, the poor experience of VR game equipment, the physical vertigo caused by VR equipment, and the technical interface between education design and game development are also challenges affecting the effectiveness of VR games.

### 1.3 Purpose of the study

Meta-analysis is a quantitative review method that aggregates and analyses experimental results from multiple independent studies to obtain more comprehensive and accurate conclusions. This study used meta-analysis to obtain relevant literature and corresponding effect size data that met the research objectives of this study in four stages: literature search, screening, assessment, and data extraction. For the definition of the research objectives, we followed the PICO framework (Higgins et al., 2019). The framework is a commonly used problem construction tool in medical research and clinical practice, and PICO stands for the four key elements: the population of patients with the problem, the intervention or treatment method, the comparison group, and the outcome. This study drew on this framework to identify the research objectives, select a student population to study, use VR science game teaching or learning as an intervention, compare it to learning or teaching without the use of VR science games, and ultimately measure the learning outcomes of the students. Next, CMA 3.0 software was used to conduct main effects analysis and moderated effects analysis on the data to reveal the effects of VR science teaching games on students’ learning outcomes. In addition, the reliability of the conclusions drawn from the study was ensured by testing the study for heterogeneity and publication bias.

The rapid development of VR technology has led an increasing number of scholars to explore the connection between cognitive processes, user behavior and environmental design in embodied environments. There is still much room for exploring the learning effect analysis and strategy design of VR science games. Therefore, based on embodied cognition theory (ECT), this study reviewed empirical research papers on VR science education games from the perspective of embodied cognition, used meta-analysis to analyze the literature, and proposed a design strategy of VR science education games from the perspective of embodied cognition, which is of great significance for improving the effectiveness of VR science education games. The research questions for this study include the following:

1. Can VR science games enhance student’s learning outcomes from an embodied cognition perspective?
2. How do moderating variables such as teaching content, game interaction, immersion mode, discipline, academic level, and intervention time affect learning outcomes?
3. How can teaching principles and strategies for VR science education games be designed?

## 2 Methods

### 2.1 Search strategy

In this study, the process of study selection strictly followed the guidelines outlined in the Preferred Reporting Items for Systematic Reviews and Meta-Analyses (PRISMA 2020) (Page et al., 2021). The datasets in the core journals of the China National Knowledge Infrastructure (CNKI) and Web of Science (WOS) databases were used as the main sources of the literature samples, spanning from December 2010 to July 2023. In the last decade, the amount of relevant literature has continued to increase as VR and science education have become widely used and highly regarded. Therefore, this time span can provide enough research information to support in-depth exploration and analysis of VR science education games. At the same time, CNKI is the largest continuously updated Chinese academic literature database. Web of Science is the premier research platform for information in the hard sciences, social sciences, arts, and humanities and the world’s most trusted publisher-independent global citation database. Together, the two cover the globe and have rigorous data selection criteria to ensure high quality and accuracy.

The keywords for the literature search include (1) VR-related keywords, including “virtual reality,” “virtual simulation,” “simulation experiments,” “virtual learning environment,” “HTC Vive,” “3D,” “somatosensory” and “experiential learning”; (2) science education keywords, including “science education,” “science,” “experimental education,” “geography,” “physics,” “chemistry” and “biology”; and (3) educational game-related keywords, including “educational game,” “game design,” “game-based learning,” “game environment” and “serious game.” HTC Vive devices have a high user acclaim and a wide range of people using the brand’s products (An et al., 2018; Bellei et al., 2018), so HTC Vive was chosen as one of the keywords for the literature search. Searches were conducted using combinations of logical operators. After searching the domestic and international databases, a total of 3,794 studies meeting the criteria were found, including 215 studies from the Chinese literature and 3,579 studies from the English literature.

### 2.2 Eligibility criteria

**Figure 1:**
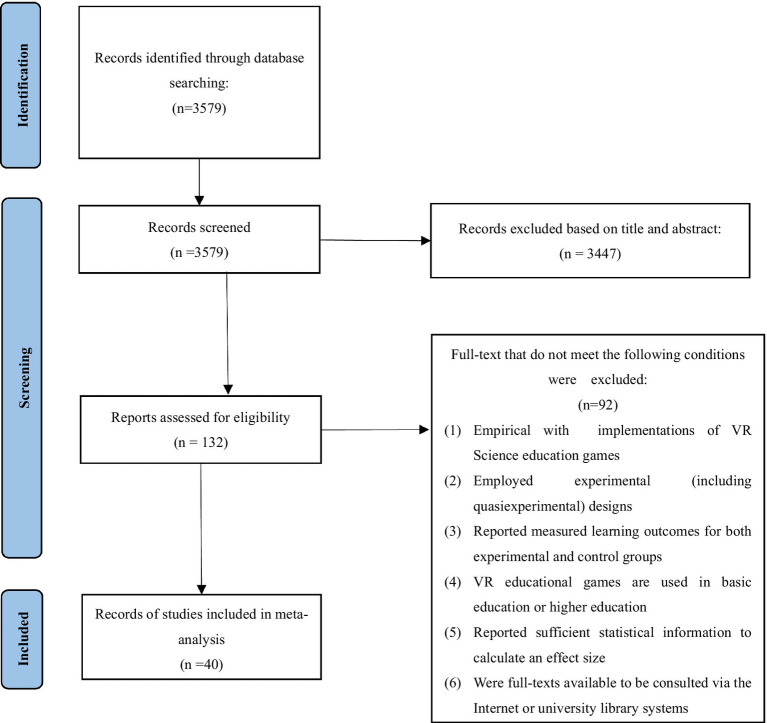
PRISMA. Flowchart for the selection process of the studies.

The flow of literature screening is shown in Figure 1. The inclusion criteria for this study were as follows: (1) included literature had to be journal articles, dissertations, or conference papers, excluding review studies; (2) studies needed to be empirical and involve the implementation of VR science education games; (3) the research subjects had to be students in both basic and higher education; (4) the research method had to be experimental or quasi-experimental, comprising experimental and control groups, with the experimental intervention focused on the utilization of VR science education games for knowledge or skill acquisition; (5) the studies had to report measured learning outcomes for both experimental and control groups, encompassing variations in learning and teaching with VR or non-VR education games. The learning outcomes of students were typically assessed through methods such as examination results, surveys, and practical assessments; and (6) full texts of the studies needed to be accessible. Following these criteria, 40 papers (representing 43 effect values) were ultimately included for subsequent data extraction and analysis, and all included literature had undergone peer review.

### 2.3 Data coding

In this study, the meta-analytic coding variables were categorized into descriptive and moderating variables. The descriptive variable factors included article title, year of publication, and author information. The moderating variables were also extracted based on embodied cognition theory, game design theory, and instructional system design theory. The coding table of moderating variables is shown in Table 1.

**Table 1:** The coding scheme.

Moderator variable	Type
Teaching content	Knowledge transfer Skills training Emotional experience
Game interaction	Active interaction Passive interaction
Immersion	Desktop immersion Somatic immersion
Academic discipline	Science Language Engineering Medicine
Segments	University Secondary school Primary school
Experimental intervention time	Long-term interventions Short-term interventions

First, Bujak et al. (2013) proposed the classic DMC game design theory, which believes that a game system should contain three elements: components, mechanics and dynamics. Game components are elements designed around the game content. Game mechanics constitute the interactive process between humans and machines that drives the progression of the game, encompassing fundamental level settings and plot advancements. Game dynamics serve as the core elements of the game system, establishing a connection with the psychological needs of the user, creating a gaming scenario, and promoting cognitive and emotional development in the player. Game design theory summarizes the general systemic components of a game, which in educational games can be represented as teaching content. With reference to the basic elements of the general instructional system design process proposed by Professor He (2016), the content of teaching is divided into knowledge transfer, skill training and emotional experience. Knowledge transfer refers to the process of teaching and transferring the knowledge content of a specific field to learners, focusing on theoretical knowledge, such as scientific principles, historical events, and mathematical formulas. Skill training develops learners’ ability to master certain practical operations or problem solving in a specific field through practice and exercises, such as experimental operations and handicrafts. Emotional experience creates an emotional connection with the learning content by creating a game situation, such as motivation, interest in learning, and sense of learning efficacy.

Second, the theory of embodied cognition suggests that the mind is embodied (Fan and Zhao, 2020), and humans interact with the objective world through the application of physical activities and sensory organs (seeing, hearing, touching, and smelling), which are classified as active and passive interactions. Accordingly, this study divides VR science game interaction into active and passive interaction. Active interaction occurs when the user actively interacts with the virtual environment through various body movements or verbal commands, such as moving, rotating, jumping, selecting options, answering questions, and performing operations. In this process, the user is the initiator of the interaction, and the process of interaction depends on the subjective decision of the user. Passive interaction, on the other hand, implies that the game system automatically presents information to the user or guides the user to perform operations, such as answering questions, listening to voices, watching videos, etc., in accordance with the predefined scenarios and plots and that the user’s operations mainly consist of observation and perception, with occasional simple reactions according to the system’s prompts.

Third, the theory of embodied cognition emphasizes the “embodiedness” of cognition (Zhang, 2016), and the generation of new knowledge requires the effective interaction of the cognitive object, the perceiving subject and the environment. In virtual reality games, the commonly used construction methods are desktop VR and immersive VR. Accordingly, this study divides VR scientific and educational game immersion methods into two categories: desktop immersion and somatic immersion. Desktop immersive VR usually uses devices such as computers or game consoles to present a virtual environment on the computer screen, and users interact with the virtual world through keyboards, mice, gamepads, or other similar devices. Physical immersive VR experiences use devices such as head-mounted displays (HMDs), motion trackers, and gloves to fully immerse the user in the virtual environment. This type of immersion allows users to interact more naturally and intuitively in the virtual world, for example, through gestures, head movements and walking.

Finally, user characteristics and information features are pivotal considerations when designing an informational environment (Bujak et al., 2013). Therefore, this meta-analysis concurrently examines whether the impact of VR educational games on student learning outcomes varies with differences in students’ academic stages, the technical subject applied, and the duration of the experimental intervention. The learner segment can be divided into primary, secondary and tertiary levels. Science education aims to help young people master the basic knowledge and skills of the natural sciences, learn the scientific method and experience the process of scientific enquiry. Since science, language, engineering, medicine and other disciplines require the use of certain scientific knowledge and methods, VR games can be used in fields related to these disciplines. If participants used the VR science game several times, it was considered a long-term experimental intervention, and conversely, if they used the VR science game only once, it was considered a short-term experimental intervention.

### 2.4 Statistical analysis and data synthesis

The aim of this study is to assess the impact of VR science and educational games from the perspective of embodied cognition on students’ learning outcomes based on experimental data from the literature, such as the mean, standard deviation, and sample size, to calculate the effect size and select Cohen’s d index as the ES statistic (Cohen, 1988). According to the effect value standard proposed by Cohen (1988), we considered Cohen’s d values less than 0.2 as small effects, Cohen’s d values between 0.2 and 0.8 as medium effects, and Cohen’s d values higher than 0.8 as large effects.

In addition, the *I*^2^ statistic test was used to test for heterogeneity in this study. According to Higgins et al. (2003), When the *I*^2^ statistic was <25%, it was considered that there was low heterogeneity among the studies; when 25% < *I*^2^ < 50%, it was considered that there was moderate heterogeneity among the studies; and when *I*^2^ > 50%, it was considered that there was high heterogeneity.

Sensitivity analyses were used to assess the robustness of the studies and to determine whether any particular study accounted for a significant proportion of the heterogeneity. Subgroup analyses were conducted based on moderating variables such as instructional content, game interaction, immersion style, discipline, academic level and duration of experimental intervention. To accurately test for publication bias, this study combined funnel plots and Begg’s test.

## 3 Results

### 3.1 Meta-analyses

**Figure 2:**
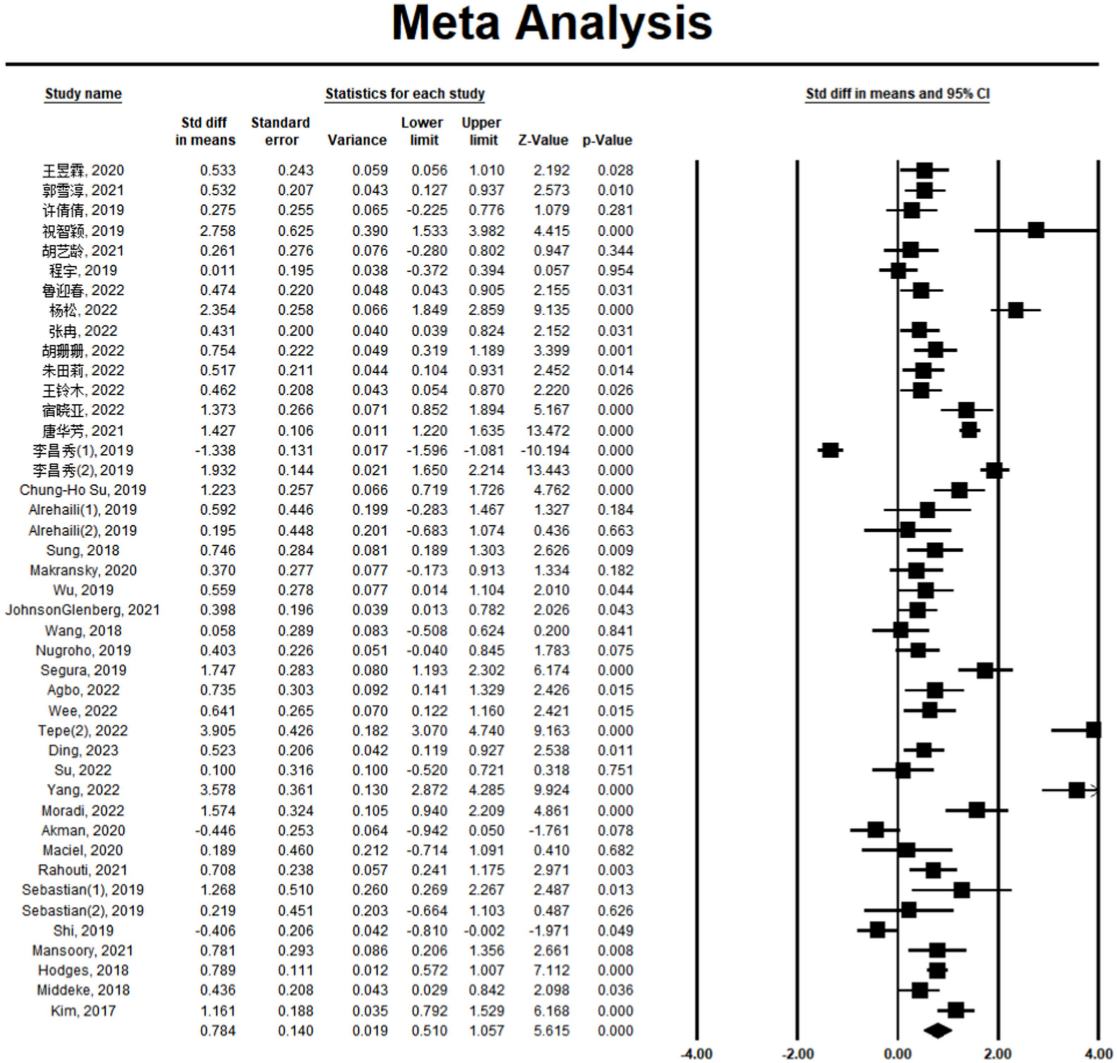
Forest plot of the effect size of VR science and education game intervention on learning outcomes. CI, confidence interval.

The results of the sample heterogeneity test showed that Q = 666.844, *p* < 0.001, and *I*^2^ = 93.702, indicating that the study combined effect values fall into highly heterogeneous intervals, and the data should be analyzed using the random effects model. The combined effect size (ES) of the VR science teaching game under embodied theory is 0.784, and the *p* value is <0.001, which reaches a statistically significant level, indicating that the VR science teaching game has a significant positive effect on students’ learning. A forest plot of the variable learning outcome can be seen in Figure 2.

### 3.2 Sensitivity analyses

Sensitivity analyses were used to verify the reliability of the results due to significant heterogeneity (*I*^2^ > 75%). When any study was removed from the model, the result of the significant effect of the VR science game on the learning effect of the exam was unchanged in the model (SMD = 0.784, 95% CI: 0.51–1.057). Thus, the results indicated that the findings for learning outcomes are robust.

### 3.3 Subgroup analyses

To investigate which moderating variables affect learning outcomes, this study coded for content, game interaction, and immersion mode. The results indicated that skill training VR science and education games (SMD = 1.091; 95% CI 0.375–1.806; *p* < 0.001), active interaction VR science and education games (SMD = 0.849; 95% CI 0.366–1.332; *p* = 0.001), somatic immersion VR science and education games (SMD = 0.895; 95% CI 0.585–1.206; *p* = 0.001), engineering disciplines (SMD = 1.54; 95% CI 0.916–2.178; *p* < 0.001), university academic disciplines (SMD = 1.005; 95% CI 0.288–1.722; *p* = 0.006) and short-term interventions (SMD = 0.846; 95% CI 0.538–1.154; *p* < 0.001) had more significant effects on learning outcomes, as shown in Table 2.

**Table 2:** Subgroup analyses based on study intervention characteristics.

Subgroup analyses				
	Number of studies	Effect size (95% CI)	*I*^2^	*p*
Teaching content Q(2) = 2.76; *p* = 0.252				
Knowledge transfer	26	0.663 (0.422–0.904)	85.165	0.000
Skills training	13	1.091 (0.375–1.806)	97.536	0.003
Emotional experience	4	0.476 (0.189–0.762)	0.000	0.001
Game interaction Q(1) = 0.325; *p* = 0.569				
Active interaction	23	0.849 (0.366–1.332)	95.895	0.001
Passive interaction	14	0.69 (0.435–0.945)	85.176	0.000
Immersion Q(1) = 1.116; *p* = 0.291				
Desktop immersion	15	0.572 (0.058–1.086)	95.974	0.029
Somatic immersion	28	0.895 (0.585–1.206)	90.969	0.000
Academic discipline Q(3) = 10.175; *p* = 0.017				
Engineering	9	1.54 (0.916–2.178)	91.983	0.000
Science	25	0.63 (0.388–0.873)	85.321	0.000
Medicine	6	0.452 (−0.789–1.692)	98.28	0.475
Language	3	0.435 (0.166–0.705)	0.000	0.002
Segments Q = 1.159; *p* = 0.56				
University	15	1.005 (0.288–1.722)	96.878	0.006
Secondary schools	12	0.759 (0.404–1.115)	83.541	0.000
Primary school	16	0.602 (0.027–1.922)	89.793	0.000
Experimental intervention time Q = 2.331; *p* = 0.127				
Short-term intervention	37	0.846 (0.538–1.154)	94.317	0.000
Long-term intervention	6	0.424 (−0.022,0.87)	80.796	0.062

### 3.4 Publication bias tests

**Figure 3:**
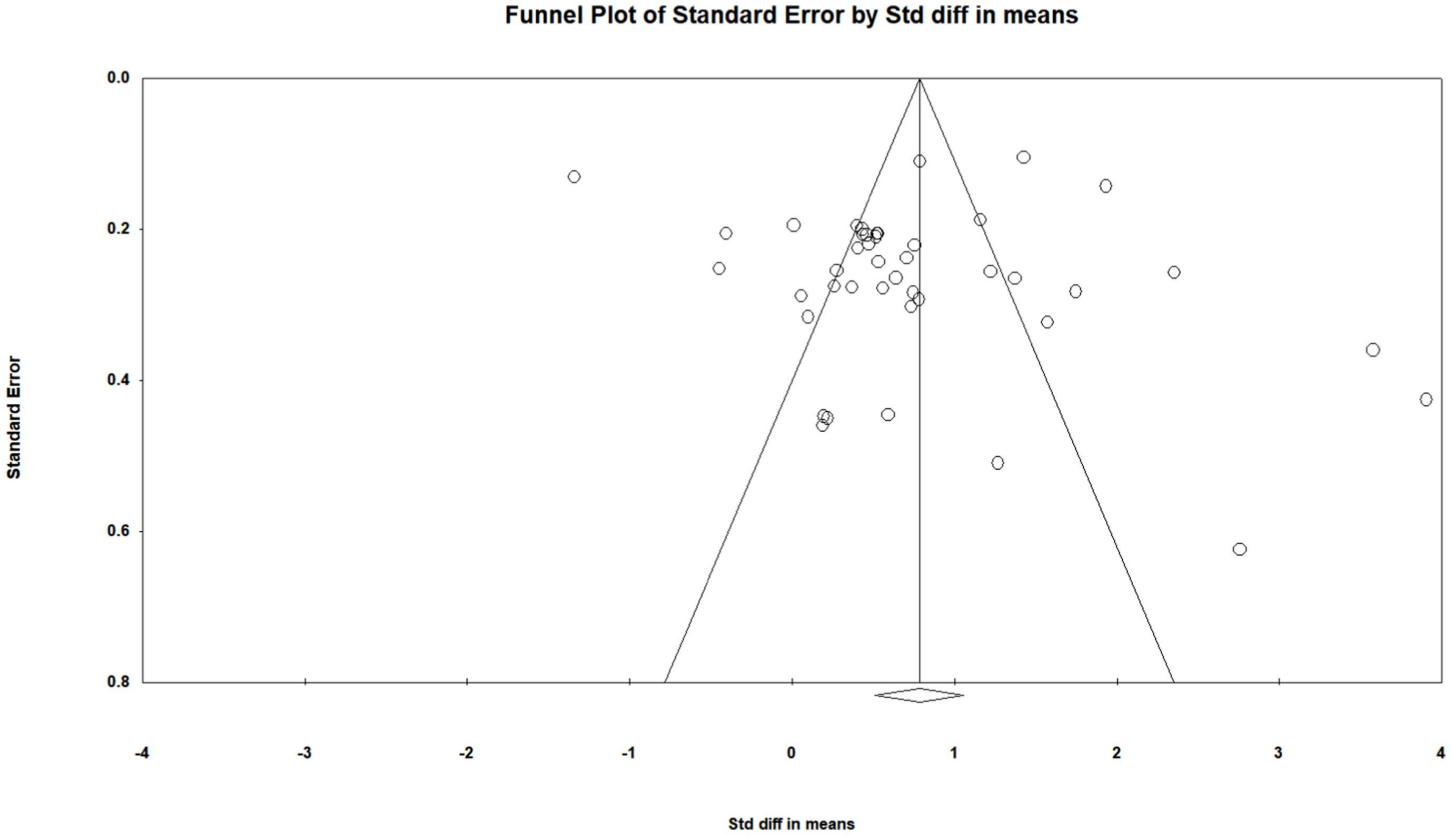
Literature analysis funnel plot.

To ensure the accuracy of the meta-analysis results, a publication bias test of the selected literature was also needed. Publication bias was tested by funnel plots and Begg’s rank correlation test. The funnel plot data were symmetrically distributed with 0.784 as the axis, and the Z value of Begg’s test result was 1.538 < 1.96, *p* = 0.124 > 0.05, which indicated that the literature publication bias was within an acceptable range (see Figure 3).

## 4 Discussion

In terms of teaching content, the learning effect of skill training VR science education games is remarkable. On the one hand, because the design of the skills training class science education game focuses on scientific practice, the value of the game is shifted from understanding scientific knowledge to experiencing the essence of practice, which is more in line with the reality of the learning process (Zhu, 2020). On the other hand, the repetitive experiments provided in skill training VR games reduce learners’ nervousness when they face unfamiliar experiments (Xiang et al., 2021). In contrast, knowledge transfer VR games are a reproduction of declarative knowledge, and in embodied VR games, learners have a stronger sense of physical control, and the known challenges of the game and the unknown knowledge of the learning process tend to decrease the learners’ enthusiasm for learning, resulting in more interest in the game than in learning and a decline in the efficiency of learning instead of an increase (Xia, 2014).

Moderation analyses of game interactions showed that active-interaction VR science and education games were better than passive-interaction VR science and education games for learning, which is the same as the findings of Lindgren et al. (2016). The reason for this is that active interaction VR science games provide learners with an active communication and feedback process, whereas in passive interaction VR science games, learners tend to observe others’ behaviors with knowledge, thus obtaining an alternative experience with a very limited level of knowledge understanding and mastery. The level of knowledge understanding and mastery is very limited (Xu et al., 2022).

The moderated analysis of VR immersion modalities showed that somatic immersion in VR science and education games had a more significant impact on the improvement of student learning. Desktop immersive VR science education games can increase learners’ cognitive engagement and make them more willing to work hard to understand knowledge, but somatic immersive VR science education games provide students with a more engaging way to learn. Research has shown that embodied immersion VR games combine cognitive and physical activities under embodiment theory, which can lead to more exercise in executive functions such as cognitive flexibility and working memory by increasing the intensity of learners’ cognitive engagement in movement (Gai et al., 2021).

Moderation analyses of technology application disciplines show that VR games combined with different disciplines lead to significant differences in student learning outcomes. In applied disciplines such as science, engineering and language, VR games significantly enhance students’ learning outcomes, and the features of immersive learning experiences and hands-on and experimental opportunities enable students to understand and apply their subject knowledge in greater depth, leading to better learning outcomes. However, in the medical field, the impact of VR games on student learning outcomes is not significant. Knowledge and skills in the medical field are usually complex and require a high degree of realism and accuracy, and although VR technology can provide an immersive experience, the current state of the art may make it difficult to fully simulate the complex situations in medical practice (Fan and Sun, 2018; Hao et al., 2022); therefore, the learning effect on the medical field may be relatively low.

Moderation analyses of the students’ academic segments showed that VR science and education games had a significant impact on the improvement of students’ learning outcomes in all academic segments. Students’ learning is more effective, especially at the university level. Older users are more experienced in using IT systems than younger users, have a mature capacity for independent learning (Yang et al., 2019) and have mature independent learning skills. They are better able to adapt to and utilize the learning opportunities provided by VR science games and thus demonstrate better learning outcomes.

Finally, the analysis of the moderation of the experimental intervention time shows that the VR science and education game significantly improves the learning effect of students in the case of short-term intervention. Short-term interventions can help students master knowledge and skills more quickly and show better learning outcomes in a short period. This may be due to the influence of the novelty effect, which affects the cognitive load level of an interactive technology when it brings novelty to the user. However, the impact of the novelty effect gradually diminishes as users gradually become familiar and adaptive to the application of VR technology (Zhang and Yao, 2023). At the same time, short-term interventions can stimulate students’ interest and motivation and provide focused and efficient learning opportunities. For long-term interventions, more factors need to be considered, such as students’ motivation, personal characteristics and learning environment. In addition, long-term interventions may face issues such as students’ adaptation to the intervention and fatigue.

## 5 Design principles and strategies

### 5.1 Design principles of a VR science game

The application disciplines, learner backgrounds and learning time of VR science education games are variable and different from person to person, which requires us to fully consider the needs and learning goals of learners from different backgrounds when designing games. As the core elements of game theory and embodied cognition theory, pedagogical content, game interaction and immersion are crucial to the learning effect and experience. However, knowledge transfer, passive interaction, and desktop immersion in VR teaching often have difficulty achieving the ideal teaching effect, so for the design of VR science education games, we need to follow some specific teaching principles and strategies to enhance the learning effect and experience of learners.

#### 5.1.1 Experiential learning principles

Kolb (1984) proposes that experiential learning involves four stages: concrete experience, observation and reflection, abstract conceptualization, and active experimentation. These four stages operate in a spiral-like, ascending manner within the human learning process. Experiential learning refers to learning through direct experiences in the context of learning (Kwon, 2019). It encourages learners to immerse themselves in practical, hands-on experiences, allowing them to gain knowledge by doing rather than just passively receiving information. This approach places the learner at the center of the learning process, fostering a deeper understanding and retention of concepts.

#### 5.1.2 Proactively explore principles

VR breaks the traditional classroom teaching thinking of teachers lecturing and students listening to lectures, giving them more opportunities to explore and learn and promoting students’ subjective and active learning (Liu et al., 2016). Proactively exploring encourages students to take an active role in discovering knowledge within the game environment. Through trial and error, students are empowered to independently explore and uncover new information and skills. This not only ignites curiosity and motivation but also cultivates their problem-solving capabilities.

#### 5.1.3 Immediate feedback principle

VR can be used for real-time data monitoring, graphical demonstration of teaching process indicators, and quantitative indicator assessment to identify problems and explore improvement options to enhance the quality of teaching (Gao et al., 2021). The immediate feedback principle ensures that students receive prompt and clear responses to their actions within the game. This allows them to understand the consequences of their actions and make necessary adjustments to their learning strategies and behaviors. Timely feedback is crucial for reinforcing learning and guiding the learner’s progress.

#### 5.1.4 Contextualized learning principles

VR technology can provide rich perceptual cues as well as multichannel (e.g., auditory, visual, tactile, etc.) feedback to help learners transfer what they have learned in virtual contexts to real life and to meet the needs of contextual learning (Huang and Liaw, 2011). VR-based games, especially role-playing serious games (RPGs), can promote learning through the simulation of various educational scenarios (Alrehaili and Al Osman, 2022). Contextualized learning emphasizes learning within specific virtual environments, making educational content more vivid and concrete. By situating learning within relevant contexts, students can better comprehend and remember the knowledge presented. This principle enhances the application of knowledge in practical situations.

#### 5.1.5 Step-by-step principle

The step-by-step principle involves breaking down the learning process into distinct stages, each with clear learning objectives and requirements. The design of educational games should pay attention to the knowledge level of students, follow the principle of from shallow to deep, step by step, and be suitable for the age and psychological characteristics of the players (Tao and Yan, 2008). This structured approach helps students gradually build skills and abilities over time, ensuring a steady progression in their learning journey.

#### 5.1.6 Human-computer interaction principles

Human-computer interaction principles pertain to the design of user-friendly, intuitive, and easy-to-use interactions between the user and the virtual environment or interface (Xiao and Guo, 2012). This ensures that users can naturally and efficiently operate and interact within the VR environment, enhancing their overall learning experience.

**Figure 4:**
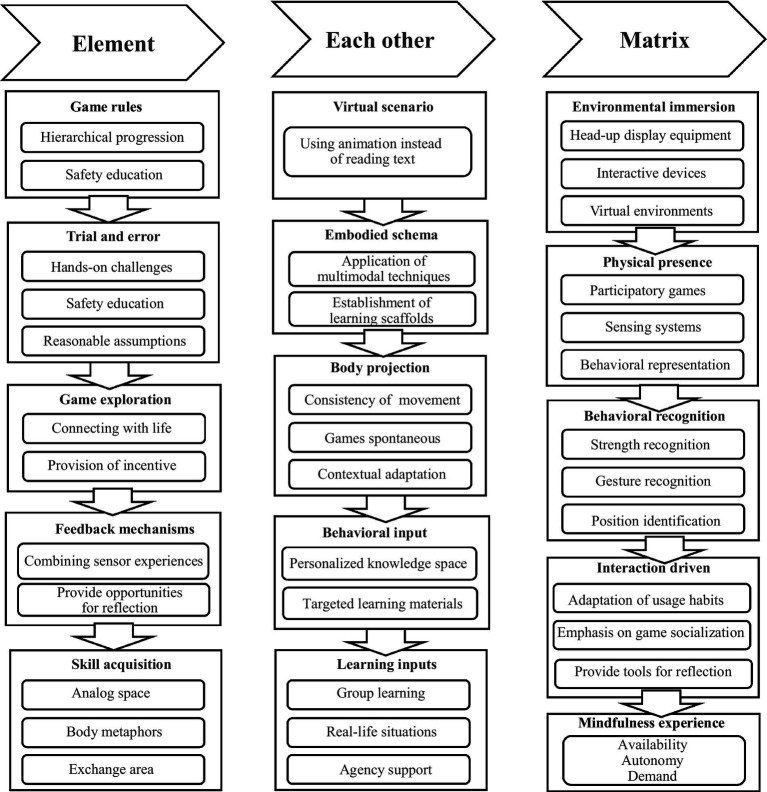
Design strategy of a VR science game based on embodied cognition theory.

In the design of VR educational games, the principles of experiential learning, active exploration, and immediate feedback are essential and nonnegotiable. They have a direct bearing on the educational efficacy and user experience of the game. Conversely, principles such as contextualized learning, step-by-step progression, and human-computer interaction can be considered supplementary guidelines in the design process. Although not obligatory, they can be flexibly adjusted according to specific circumstances and design objectives. According to the above principles, the VR science education game design strategy based on embodied cognition theory is proposed in order to be able to enhance students’ skill training, active interaction ability and immersion experience, as shown in Figure 4.

### 5.2 Skills training content design 5-stage strategy

Based on the results of meta-analyses, the learning effect of skill training VR science education games is remarkable. The creative content of VR science education games should focus on skill training. Embodied cognition emphasizes the “embodiedness” of cognition, and the generation of new knowledge requires effective interaction between the cognitive object, the perceiving subject and the environment. From the perspective of embodied cognition, VR games allow students to return to the same tasks repeatedly during the learning process through the interaction between the body and the game and enhance the deliberate practice of a certain skill through continuous experimentation and trial-and-error. A 5-stage strategy is included: game rules – embodied trial and error – game exploration – feedback mechanism – skill acquisition.

#### 5.2.1 Game rules

Complex and open virtual spaces are prone to problems such as overly dispersed learning behaviors and uncontrolled learning processes. Setting the rules of breaking through from easy to difficult can enhance the consistency of learning objectives and game behavior. (1) Level progression. Game materials were provided progressively according to the learning content. For example, when users are making rockets, if they have not completed the first level of rocket making, the game does not provide the second level of rocket equipment. (2) Safety education. Provide for dangerous behaviors in the game. For example, Johnson-Glenberg (2018) set the first level in the evolution game for learners to distinguish poisonous butterflies and to end the game if they accidentally ate poisonous butterflies.

#### 5.2.2 Trial and error with the body

In science education, “mistakes” are considered excellent learning materials. Using trial and error as a vehicle for teaching content can help each student identify his or her own problems. Liu et al. (2022) classified the types of errors into material processing, observation and statistics, theory construction and knowledge dissemination. The design of embodied VR science teaching games can be used to (1) provide learners with hands-on game challenges of experimental instrument processing and experimental condition judgment so that learners can continuously experience trial and error; (2) provide learners with optional analysis tools to try to analyze the experimental results and reflect on the experimental problems; and (3) provide game options to guide students to make reasonable assumptions and correct attributions.

#### 5.2.3 Game exploration

The process of game exploration is the process of learners’ active learning. When conducting biological observation experiments, it was found that by setting up tasks to break into the game, students had the opportunity to get to know the Sichuan snub-nosed monkey from different perspectives and were able to easily determine the problems of their own operation (Zhang and Zhang, 2013; Zhou et al., 2020). Therefore, attention should be given to relevance and incentives in the design of inquiry activities: (1) Connect the objective facts with the game situation. For example, in the IVR-Honeybee game, to transport honey safely back to the hive, learners must correctly identify and avoid natural enemies. (2) Provide game rewards. The game should encourage learners to consistently complete the experiment by setting up game leaderboards, points and props, which can improve students’ emotional experience, stimulate students’ motivation and interest in learning (Zagalo et al., 2005; Chen and Hsu, 2020; Wang et al., 2023), and achieve the effect of developing students’ skills.

#### 5.2.4 Feedback mechanisms

Meaningful play experiences occur when there is a discernible and purposeful relationship between play actions and output outcomes (Tekinbas and Zimmerman, 2003). For example, the process of graphene conductivity occurrence is simulated by having learners touch graphene with an electric current touch, replacing the visual experience with a tactile sensation (Zhai et al., 2021). Therefore, different sensory interactions can be designed as game feedback. Importantly, in game-based learning, in addition to the stimulus-feedback linkage, learners should be provided with opportunities for reflection. Short, immediate and actionable tests and questions can deepen learners’ understanding of scientific steps during experiments.

#### 5.2.5 Skill acquisition

Skill acquisition is a process of imitation, epiphany, and innovation (Qiu and Qiu, 2020). Based on this, the links of the game should be designed to conform to the rule of providing simulation space, body metaphor and social interaction. (1) A simulation space should be provided, as well as the guidelines needed for skill acquisition. This includes building a virtual laboratory, learning videos, material kits, and using game components such as music rhythms, signposts, and blinking for game prompts. (2) Body metaphors should be applied to mobilize the connection between brain cognition and body iconography. To introduce users to the principle of friction-generated electricity, Segal (2011) first played an animation for the viewer of the build-up of electric charge after friction on the hand and then had the learner move his or her foot back and forth on the floor and monitor the movement with the Kinect sensor, which showed the build-up of electric charge on a virtual display. (3) Experiences between groups in the real world should be shared. At the end of the game, students summarize and reflect on the principles learned through group work and consultation and exchange (Zhao and Zhong, 2021).

### 5.3 Five-stage strategy for active human-computer interaction design

Moderation analyses of game interactions showed that active-interaction VR science and education games were better than passive-interaction VR science and education games for learning. The theory of embodied cognition can guide us in designing active-interactions. Embodied cognition suggests that creating game interaction pathways that refer to real scientific inquiry processes can resonate with learners, point them in the direction of thinking, and thus increase engagement in learning. Embodied games view the human body as a mediator for perceiving learning content and promote embodied learning by setting up learner-initiated behaviors of observation, simulation, and dialog (Fan and Zhao, 2020). The human body is viewed as a mediator of perceived learning content. Accordingly, the human-computer interaction design strategy of VR educational games from the perspective of embodied cognition can be summarized as follows: virtual scene – embodied schema – body projection – behavioral input – learning input.

#### 5.3.1 Virtual scene

The construction of the game scene is the condition guarantee for the realization of embodied interaction. The design of the virtual scene should be in line with the needs of game exploration activities and the use of graphics or animation and other media instead of text reading to enhance human-computer interaction and reduce the transmission of text knowledge. On the one hand, focusing on text for a long time in a VR headset can cause eye pressure and discomfort (Hostetter and Alibali, 2008). For example, in the butterfly observation game designed by Johnson-Glenberg (2018), the animation of the scene in which the butterfly emerges from the cocoon is used to replace the textual instructions. On the other hand, too much text knowledge will kill the enthusiasm of learners, and the learning efficiency will decrease instead of increase.

#### 5.3.2 Embodied schema

Body schema is a perceptual-motor system ability. It is an individual’s unconscious invocation of body movements and postures (He, 2009). For example, when we want to take a cup to drink, our hand will move toward the cup accurately and automatically. The body metaphors designed in the game content need to interact with and be manipulated to be truly transformed into body schemas (Shang et al., 2008). The body metaphor designed in the game content needs to be transformed into a body schema through interaction and actual manipulation. To facilitate the transformation of schema, the interaction design can (1) apply multimodal technology to experience the cognitive processing process. Through data evaluation, expression recognition, and sensor tracking, multimodal information can be collected, processed, interacted with, and outputted and then used in the game in the form of contextual stories and simulation experiments to form body schemas (Johnson-Glenberg, 2018). (2) To establish learning scaffolding, complex cognitive processes need scaffolding to provide assistance. Each challenge should provide an unfamiliar variable, and learners need to keep breaking through to gradually master scientific principles.

#### 5.3.3 Body projection

Embodied cognition theory suggests that the body mediates the perception of learning content and that the learner’s active acts of observation, simulation, and dialog can promote embodied learning (Song and Cui, 2021). Therefore, when designing game interactions, attention should be given to the consistency, spontaneity, and interaction of movement and interaction. (1) Consistency: Learners’ gestures and movements should be correctly mapped to the learning content. For example, when drawing a circle, the elbow should be rotated, not panned or moved forward. (2) Spontaneity: The learner is guided to a certain physical behavior. For example, learners are asked to predict the shape of a line when a ball is thrown horizontally and express it in a movement and then present the corresponding line in the display. (3) Adaptability: Different learning styles and age groups of learners have different behavioral characteristics (Shang et al., 2008; Kalyuga, 2009), so it is necessary to match the adapted body schema according to the characteristics of the learners.

#### 5.3.4 Behavioral inputs

In addition to constructing body metaphors and body schemas, the game interaction needs to take learners’ personalized behaviors into account. For example, according to the learner’s preset shortcuts, a personalized operation interface and private learning notes are set up. According to the difficulty of the game level, we can provide instant communication and discussion forums. Speech recognition technology can take the input of the learner’s voice and analyze it in order to provide timely emotional feedback and interaction through the expressions, gestures and vocal tones of appropriate virtual characters (Chen and Hsu, 2020). At the same time, the game platform can also analyze learning behavior data and provide adaptive materials and personalized learning maps based on learners’ game completion.

#### 5.3.5 Learning inputs

Learning engagement is a positive, fulfilling mental state for students (Schaufeli et al., 2002; Fang et al., 2008). To enhance learners’ engagement in learning, from the interaction perspective, on the one hand, more opportunities should be provided to play games in groups. Research has shown that playing games with age-appropriate new users can stimulate the brain to reinterpret past experiences (Oculus, 2018). On the other hand, the use of authentic cultural contexts can improve learning engagement. For example, the game “Alien Health” creates an alien malnutrition puzzle based on food and cultural practices, where children have to choose the correct food combinations within a time limit.

### 5.4 5-stage strategy of somatic immersive environment design

The moderated analysis of VR immersion modalities showed that somatic immersion in VR science and education games had a more significant impact on the improvement of student learning. The theory of embodied cognition suggests that an embodied immersion experience will have a positive impact on the cognitive effects of learners. Based on embodied theory, VR science education games place higher requirements on the environment immersion experience with technical support, and experimental learning involves body perception, material technology, and spatial and temporal relations, which interact with each other to jointly contribute to embodied cognition. The environmental immersion design of VR science education games from the perspective of embodied cognition contains a 5-stage strategy: environment construction – body presence – behavior recognition – interaction drive – mental experience.

#### 5.4.1 Environmental constructs

Influences on immersive game-based learning environments include the physical environment, physical presence, and mindfulness experiences (Jiang and Shiyan, 2022). The physical environment is the external support for creating student immersion and consists of headset devices, interactive devices and virtual environments. (1) Gaming headset devices. VR games require high processor performance and interactivity, so external displays with independent screens (Oculus Rift) and all-in-one displays without data cable constraints are commonly used (Li et al., 2018). (2) Interactive devices. Interaction devices are sensor controllers (Kinect, Leap motion) provided to meet the needs of users interacting with the game action. (3) Virtual environment: VR games take place in a VR network environment and are usually built using more advanced Unity or Unreal Engine graphics technologies to create more realistic, immersive virtual environments (Foxman, 2019; Wiesing et al., 2020).

#### 5.4.2 Physical presence

VR science and education games based on embodied theory place higher demands on physical immersion. During the game, technical sensitivity and realism, such as closing movements and haptic feedback, are important parts in creating a highly embodied state for students (Pasch et al., 2009). Zheng et al. (2021) developed a virtual physical space supporting force feedback based on touch devices, where learners can feel the friction of different materials (wood, quartz, and glass) through force feedback devices. The design of embodied games should take physical participation into consideration, with learning activities as the main line, and mobilize the participation of learners’ body perceptual-motor systems (movements, gestures, expressions, speech, etc.) through playing, manipulating, observing and other methods. With the help of somatosensory devices, wireless sensors and other accurate and real-time collection of learners’ body behavior and state information, a representation of embodied behavior is finally formed.

#### 5.4.3 Behavioral recognition

Behavior recognition (action recognition) is a class of technology that uses specific algorithms to analyze collected big data to identify, analyze and cluster human behavior. In game design, specific behavioral action-response mechanisms (strength recognition, gesture recognition, position recognition) can be set up to respond to specific learning behaviors. For example, in the VR dental simulator, through force recognition, when abnormal values are monitored, surgical results are given according to the abnormal values (Yin et al., 2021); through positional recognition, the learner is rewarded when he or she correctly places a note into the recognizable area (Lu et al., 2022). In addition, through gesture recognition, it can judge the direction of game advancement based on learners’ gestures, judge whether to force learners to go offline based on the length of their online time, and so on.

#### 5.4.4 Interaction driven

Interaction is the key to the success of VR games. The human-computer interaction process in the game replaces the teacher-student interaction behavior in the classroom (Wang et al., 2018). The interaction between teachers and students in the classroom is replaced by the human-computer interaction process. Therefore, when designing the game, we should (1) pay attention to the game’s social interaction (verbal interaction, expression and action communication), providing an environment for learners to find partners and exchange experiences, and (2) provide reflection tools to support learners in opening the knowledge management tool at any time and any place to build their personal learning maps. (3) Consider learners’ usage habits, simplify technical difficulties, and use robots or help manuals to answer learners’ usage questions.

#### 5.4.5 Mindfulness experience

The design goal of VR science games is not only to facilitate the acquisition of inquiry skills but also to give learners a good mental experience in a relaxed and natural environment. Factors affecting learners’ mental experience include the usability, demand and autonomy of the game (Jiang, 2020). (1) Usability. Children and teenagers are still developing their ability to understand abstract words. In this regard, the difficulty of the game can be reduced by setting up interactive game objects and changing the background of the game (swinging, flashing, darkening) to imply the experimental situation. (2) Autonomy. During the game, you should give learners opportunities to make their own judgments about the authenticity of information. For example, nonrole players (NPCs) can give confusing information, and learners need to judge authenticity on their own. (3) Demanding. Learning needs are crucial in game environment design. Due to the limited cost of VR game development, the game should be presented as concisely and clearly as possible and should not be too complex or fancy.

## 6 Conclusion

In the era of digital-intelligence integration, embodied learning that integrates the body and mind will bring a new learning experience. Based on the theory of embodied cognition, this study discusses the current research status and application challenges of VR scientific and educational games and then proposes design strategies for VR scientific and educational games from the three aspects of content design, interaction design and environment design. Game design from the perspective of embodied cognition still has many problems to be improved. On the one hand, the mature theory of embodied game design has not been systematically constructed. Fully mobilizing embodied perception to achieve deeper integration from behavior to cognition is both a difficulty in the current theoretical design and a key point in future exploration and practice. On the other hand, the heterogeneity of the existing game resources is high, and the repeatability of the games is low, which leads to the high cost of game development. The construction of the platform and the design of the standardized model for the VR virtual science education game is an urgent problem to be solved.

## Data availability statement

The original contributions presented in the study are included in the article/supplementary material, further inquiries can be directed to the corresponding author.

## Author contributions

XL: Conceptualization, Funding acquisition, Methodology, Writing – original draft, Writing – review & editing. RL: Data curation, Writing – original draft. ZC: Data curation, Writing – original draft. JX: Data curation, Methodology, Writing – original draft, Writing – review & editing.
